# Contributions of school context to caries on anterior teeth: a multilevel analysis

**DOI:** 10.11606/s1518-8787.2021055003929

**Published:** 2021-12-03

**Authors:** Isolda Mirelle de Lima Ferreira Prata, Érick Tássio Barbosa Neves, Larissa Chaves Morais de Lima, Laio da Costa Dutra, Fernanda Morais Ferreira, Saul Martins Paiva, Ana Flávia Granville-Garcia

**Affiliations:** I Universidade Estadual da Paraíba Programa de Pós-Graduação em Odontologia Campina Grande PB Brasil Universidade Estadual da Paraíba. Programa de Pós-Graduação em Odontologia. Campina Grande, PB, Brasil; II Universidade Federal de Minas Gerais Faculdade de Odontologia Departamento de Odontopediatria e Ortodontia Belo Horizonte MG Brasil Universidade Federal de Minas Gerais. Faculdade de Odontologia. Departamento de Odontopediatria e Ortodontia, Belo Horizonte, MG, Brasil; III Universidade Estadual da Paraíba Faculdade de Odontologia Departamento de Odontologia Campina Grande PB Brasil Universidade Estadual da Paraíba. Faculdade de Odontologia. Departamento de Odontologia. Campina Grande, PB, Brasil

**Keywords:** Adolescent, Dental Caries, prevention & control, Oral Health, Health Literacy, School Health Services

## Abstract

**OBJECTIVE:**

To investigate whether oral health literacy (OHL) and school context are associated with untreated dental caries on the anterior teeth of adolescents.

**METHODS:**

A representative cross-sectional study was conducted with 746 students aging 15 to 19 in the city of Campina Grande, Brazil. The guardians answered a questionnaire addressing sociodemographic data and the absence/presence of private health insurance. Two examiners were trained for the diagnosis of dental caries using the Nyvad criteria and the measurement of OHL using the Brazilian Rapid Estimate of Adult Literacy in Dentistry (BREALD-30) (Kappa > 0.80). Contextual aspects of the schools were obtained from the 2017 National School Census. Descriptive statistics were conducted, followed by unadjusted and adjusted robust negative binomial regression for complex samples (p < 0.05).

**RESULTS:**

The average number of anterior teeth with untreated caries was 0.95 (SD = 1.77). Among individual factors, the male sex (RR = 1.64; 95%CI: 1.24–2.16), inadequate level of OHL (RR = 2.03; 95%CI: 1.13–1.63), marginal level of OHL (RR = 1.87; 95%CI: 1.05–3.33) and not having private health insurance (RR = 1.34; 95%CI: 1.07–1.68) were associated with untreated caries on anterior teeth. Among school contextual factors, the number of students in the classroom (RR = 2.64; 95%CI: 1.78–3.93), number of public oral health services in the district (RR = 0.14; 95%CI: 0.05–0.39) and average income of the district in which the school is located (RR = 0.99; 95%CI: 0.98–0.99) were associated with the outcome.

**CONCLUSIONS:**

Sociodemographic factors, having private health insurance, OHL, and school context exerted an influence on the occurrence of untreated dental caries on anterior teeth in adolescents aging 15 to 19.

## INTRODUCTION

Dental caries is an oral health problem with a worldwide impact^1^ and is highly prevalent in adolescence (53.8%–71.7%)^2,3^. Studies addressing caries on the anterior teeth are important due to the greater esthetic impact compared to other regions of the oral cavity^4,5^ and the negative influence on the quality of life of adolescents^6^. Therefore, it is important to explore individual factors and aspects of the school context associated with untreated caries on anterior teeth, as studies of this nature could contribute to caries control strategies for adolescents^7^, which would have better repercussions in adulthood.

Oral health literacy (OHL) is an important individual oral health determinant. OHL regards the degree to which individuals process and understand oral health information and use such information when making decisions about health^8^. Thus, a high level of OHL contributes to healthy behaviors^9^, whereas low OHL levels are associated with poorer oral health outcomes, such as dental caries in adolescents^10,11^. However, no previous studies have investigated the influence of OHL on the occurrence of untreated caries on anterior teeth. Such information is important, as low OHL may be a critical indicator of oral health status in this population, since the anterior region of the oral cavity is the easiest to inspect and perform oral hygiene.

School is a space where students spend several hours per day and plays an important role in both intellectual development and adolescent behavior^12^. Therefore, characteristics of the school environment can influence oral health practices^13^. Associations between untreated dental caries in adolescents and school contextual factors, such as the number of students in the classroom, the number of dental services in the district where the school is located, and the average income of the district, should be explored, as these factors indicate standards to which adolescents are submitted and information on such factors could assist in the establishment of caries prevention policies. Thus, studies that use a multilevel approach could assist in identifying priority groups for oral health actions^7^.

The conceptual hypothesis tested here is that OHL and school contextual factors influence the number of untreated dental caries in adolescents. Therefore, this study aimed to investigate whether individual and contextual factors are associated with untreated dental caries on anterior teeth in adolescents.

## METHODS

### Ethical Considerations

This study was conducted in accordance with the ethical principles of the Declaration of Helsinki and received approval from the institutional review board of the Universidade Estadual da Paraiba (protocol number: 55953516.2.1001.5187).

### Study Design and Sample Selection

An analytical, population-based, cross-sectional study was conducted with adolescents aging 15 to 19 enrolled at public and private schools in the city of Campina Grande, Brazil. Data collection occurred between October 2016 and July 2017. Students who wore orthodontic appliances at the time of the examination, those with physical or cognitive disabilities that required special assistance, and foreign adolescents were excluded from the study.

The participants were selected through probabilistic cluster sampling in two stages (schools and adolescents). A total of 131 schools were registered with the Ministry of Education in the city. Thirty-two schools (16 public and 16 private) were selected through a random drawing with the proportional distribution of adolescents in the six administrative districts of the city. In the second stage, students were selected from each school using a simple randomization procedure.

The sample size was calculated for analytical studies with comparisons of means between two independent groups using the G*Power software, version 3.1.9.7 (Franz Faul, Universitat Kiel, Germany), adopting a 95% significance level and 80% power. Based on data from a pilot study, in which the average number of anterior teeth with untreated caries in adolescents with inadequate and adequate OHL was respectively 1.6 (SD = 1.3) and 0.7 (SD = 1.1), the minimum sample was calculated to be 394 adolescents. A design effect of 1.6 was applied to compensate for the cluster sampling procedure, resulting in 631 individuals, to which 20% was added to compensate for possible dropouts, leading to a final sample of 789 adolescents.

### Training and Calibration Exercises

Two examiners were trained for the diagnosis of dental caries using the Nyvad criteria^14^. The training and calibration steps were conducted by a dentist with expertise in the field. The theory step consisted of the study of the diagnostic criteria for dental caries. The practical step involved the examination of 50 adolescents on two occasions separated by a seven-day interval for the determination of inter-examiner (K = 0.89 to 0.90) and intra-examiner (K = 0.88 to 0.90) agreement using the Kappa statistic. Training for the administration of the Brazilian version of the Rapid Estimate of Adult Literacy in Dentistry (BREALD-30)^15^ was conducted by a researcher with a large experience in the investigation of OHL using this instrument^16^. After a discussion on the use of the instrument (theory step), the calibration step was performed using a bank of 15 videos of individuals with different degrees of OHL who answered the BREALD-30. Kappa coefficients for agreement between the examiners and the experienced researcher were 0.889 and 0.884. The agreement between the two examiners was K = 0.870 and the intra-examiner agreement was K = 0.898 and 0.871.

### Pilot Study

Prior to the data collection, a pilot study was conducted with 50 adolescents aging 15 to 19 enrolled at one private school (n = 25) and one public school (n = 25). The students were selected randomly and were not included in the main study.

### Collection of Non-Clinical Data

The individual variables collected in this study corresponded to characteristics related to the adolescents. Data on sex, ethnicity, health insurance, and family socioeconomic status were obtained from a questionnaire filled out by the guardians of the adolescents. Social class was determined using the Brazilian Economic Classification Criteria^17^, which considered the education level of the head of the household, the number of consumer goods, indoor plumbing, and living on a paved street. These criteria enabled classifying the adolescents into five economical classes (A, B, C, D, and E), with subsequent dichotomization into high (Classes A and B) or low (Classes C, D, and E) economic status.

The BREALD-30 was administered to evaluate the level of functional OHL of the adolescents. This word recognition instrument has previously been validated for use on this population^15^ and consists of 30 terms related to oral health arranged in increasing order of reading difficulty. The adolescents read each word aloud to the examiner, who attributed one point for the correct pronunciation and zero for the incorrect pronunciation. A higher number of correctly pronounced words is considered indicative of a higher level of OHL. The total scores were divided into terciles and categorized as inadequate (0–18), marginal (19–22), or adequate (23–30). The students were also asked whether they had been to a dentist at some time in life.

The contextual variables of interest were related to the school setting. The average number of students per classroom was determined based on the 2017 National School Census^18^. The number of dental services in the administrative district where the school was situated was obtained from the city’s Department of Health and the average income in the school district was determined based on data from the Brazilian Institute of Geography and Statistics (IBGE) of the city. There is no consensus in the literature on the use of these school context variables in categories, and their use as continuous data enables a more robust analysis that better reflects the behavior of the variables under analysis.

### Collection of Clinical Data

To assist the diagnosis, the adolescents performed supervised tooth brushing prior to the clinical examination. In a private room at the school, the subject remained seated in front of the examiner, who dried the dental surfaces with sterile gauze and performed relative isolation with cotton. The examinations were performed with the aid of a headlamp, mouth mirror, and periodontal probe.

The criteria proposed by Nyvad and Baelum (2018) were used for the diagnosis of untreated caries on anterior teeth. These criteria are based on caries activity and severity, classified in categories from 0 to 9. For the present study, the following codes were considered: 1 (active caries with intact surface), 2 (active caries with discontinuous surface), and 3 (active caries with cavitation). Only active caries were considered because such lesions reflect a current need for dental care and offer a more sensitive portrait of current oral health status and postponement in seeking dental services.

### Statistical Analysis

The organization of the data and statistical analysis were performed using SPSS Statistics (SPSS for Windows, version 22.0; IBM Inc., Armonk, NY, USA). Descriptive analysis was performed for the characterization of the sample.

The number of anterior teeth with untreated caries was the dependent variable and was treated as a continuous numerical variable to enable a better assessment of severity. It is important to consider that an adolescent may have more than one tooth with decay and a larger number of untreated dental caries, which indicate a poorer oral health status. Moreover, using a continuous outcome enables a better fit of the data and provides a more robust model. Unadjusted robust negative binomial regression was performed to determine individual and contextual variables associated with the outcome. This type of regression analysis was selected because the variance in the outcome was higher than the mean. Negative binomial regression enables flexibility in cases of model overdispersion and is a better fit to the data compared to Poisson models. Variables with a p-value < 0.20 were incorporated into the multilevel model.

In the first step, an unconditional (null) model was used to estimate the variability in the data and did not include the predictors. Individual variables were added to the second model (Model 2). Model 3 included individual and contextual variables. Variables with a p-value < 0.05 in the adjusted analysis were significantly associated with untreated dental caries. The goodness of fit of the models considered deviation values (–2 log likelihood).

## RESULTS

The Figure shows the theoretical framework of this study based on previous literature. The sample was composed of 746 adolescents. The non-response rate was 5.5% (n = 43). Dropouts were due to absence from school on the days scheduled for the examinations. The average number of anterior teeth with untreated caries was 0.95 (SD = 1.77) and the prevalence of teeth with untreated active dental caries was 33.5%. Table 1 shows the data for the characterization of the sample.

**Figure:**
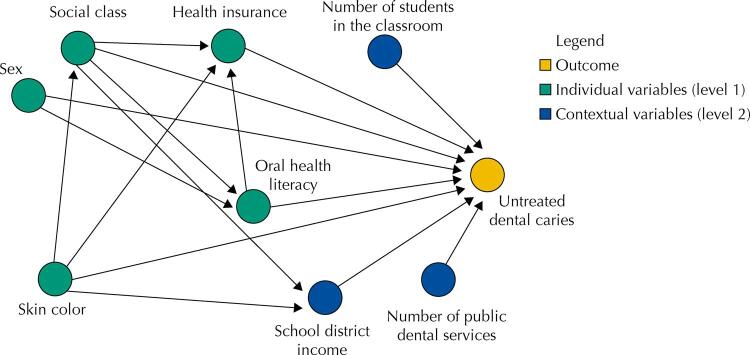
Directed acyclic graph (DAG) for the association of oral health literacy (OHL) and school context with untreated dental caries on the anterior teeth of adolescents.

**Table 1:** Oral health literacy, sociodemographic, clinical, and school context characteristics of the sample.

Variables	n (%) / Mean (SD)
Individual variables	
Sex	
Male	302 (40.5)
Female	444 (59.5)
Skin color	
Non-white	535 (71.7)
White	211 (28.3)
Social Class	
Lower	428 (57.4)
Upper	318 (42.6)
Dental visit	
No	299 (6.2)
Yes	443 (93.8)
Oral health literacy	
Inadequate	412 (55.2)
Marginal	218 (29.2)
Adequate	116 (15.6)
Health insurance	
No	474 (63.7)
Yes	270 (36.3)
Untreated dental caries	
No	496 (66.5)
Yes	250 (33.5)
Number of teeth with untreated caries	0.95 (1.77)

Contextual variables	
Number of students in the classroom	28.2 (7.39)
Public dental services in the district of the school	5.54 (1.19)
School district income	1,148.0 (668.8)

Table 2 shows the results of the unadjusted robust negative binomial regression analysis. All variables with a p-value < 0.20 were incorporated into the multilevel model.

**Table 2:** Unadjusted robust negative binomial regression for the association of individual and contextual variables associated with the number of anterior teeth with untreated dental caries.

Variables	Dental caries Mean (SD)	p	Unadjusted RR RR (95%CI)
Individual-level			
Sex			
Male	1,10 (1,83)	0.069^a^	1.27 (0.98–1.66)
Female	0,86 (1,72)		1.00
Skin color			
Non-white	0,98 (1,80)	0.518	0.90 (0.67–1.22)
White	0,89 (1,68)		1.00
Social class			
Lower	1,13 (1,91)	0.001^a^	1.59 (1.19–2.11)
Upper	0,71 (1,53)		1.00
Dental visit			
No	1,50 (2,34)	0.042^a^	1.62 (1.01–2.59)
Yes	0,92 (1,72)		1.00
Oral health literacy			
Inadequate	1,15 (1,90)	< 0.001^a^	2.43 (1.51–3.89)
Marginal	0,84 (1,73)	0.030^a^	1.78 (1.05–2.99)
Adequate	0,47 (1,16)		1.00
Health Insurance			
No	1,10 (1,95)	0.001^a^	1.59 (1.19–2.11)
Yes	0,69 (1,37)		1.00

Contextual level			
Number of students in the classroom	-	0.021	0.97 (0.96–0,99)
Public dental services in the district of the school	-	< 0.001	0.80 (0.71–0.90)
School district income	-	0.009	0.99 (0.98–0.99)

Unadjusted Rate Ratios (RR) for robust multilevel negative binomial Regression to evaluate the association of individual and contextual variables and the number of anterior teeth with untreated dental caries.

^a^ Variables included in the multivariate model (p < 0.20).

Table 3 shows the results of the adjusted multilevel analysis of the individual and contextual variables associated with untreated dental caries. The individual variables associated with the outcome (p < 0.05) in Model 2 remained associated in the final model (p < 0.05) after the adjustments for the contextual variables (Model 3). Male adolescents (RR = 1.64; 95%CI: 1.24–2.16), those with inadequate OHL (RR = 2.03; 95%CI: 1.13–3.63), those with marginal OHL (RR = 1.87; 95%CI: 1.05–3.33), and those with no health insurance (RR = 1.34; 95%CI: 1.07–1.68) had a greater number of anterior teeth with untreated caries. Contextual determinants of the school also affected the number of anterior teeth with untreated caries. The average number of students per classroom was associated with an increase in dental caries (RR = 2.64; 95%CI: 1.78–3.93). A greater number of public dental services in the district where the school was located (RR = 0.14; 95%CI: 0.05–0.39) and a higher average income in the district (RR = 0.99; 95%CI: 0.98–0.99) were protective factors for untreated caries.

**Table 3:** Multilevel negative binomial regression for the assessment of individual and contextual variables associated with the number of anterior teeth with untreated dental caries among adolescents.

	Model 1 (“null”)	Model 2		Model 3	
		
	RR (95%CI)		RR (95%CI)		RR (95%CI)
Intercept	0.64 (0.47–0.87)	p	0.22 (0.10–0.48)	p	1.40 (0.01–1.58)
Individual level					
Sex					
Male	–	0.001	1.58 (1.19–2.09)	< 0.001^a^	1.64 (1.24–2.16)
Female	-	-	-	-	-
Social class					
Lower	-	-	-	-	-
Upper	-	-	-	-	-
Dental visit					
No	-	-	-	-	-
Yes	-	-	-	-	-
Oral health literacy					
Inadequate	-	0.017	1.99 (1.13–1.52)	0.017^a^	2.03 (1.13–3.63)
Marginal	-	-	-	0.034^a^	1.87 (1.05–3.33)
Adequate	-	-	-	-	-
Health insurance					
No		0.030	1.28 (1.02–1.60)	0.010^a^	1.34 (1.07–1.68)
Yes	-	-	-	-	-

Contextual level					
Number of students in the classroom	-	-	-	< 0.001^a^	2.64 (1.78–3.93)
Public dental services in the district of the school				< 0.001^a^	0.14 (0.05–0.39)
School district income	-	-	-	0.030^a^	0.99 (0.98–0.99)
Deviance (-2loglikelihood)	26,221.17		24,247.89		24,009.74

Model 1 (“null”): presents the unconditional model; Model 2: presents individual covariates; Model 3: presents individual and contextual-level covariates.

^a^ Significance level at p < 0.05.

## DISCUSSION

The conceptual hypothesis of this study was confirmed. Individual and school contextual factors exerted an influence on the occurrence of dental caries. Male adolescents with inadequate/marginal OHL, and those without health insurance had a greater number of anterior teeth with untreated caries. Regarding the school context, the results suggest that each one-point increase in the number of public dental services in the school district and each one-point increase in school district income were associated with respective reductions of 86% and 1% in the number of anterior teeth with untreated dental caries among the adolescents. Moreover, each one-point increase in the number of students in the classroom was associated with a 2.6-fold increase in the number of anterior teeth with untreated dental caries. These data are important and can contribute to strategies for oral health promotion and the prevention of caries in this population. The findings also underscore the importance of the participation of schools in such programs.

The average number of anterior teeth with untreated caries in the present study was 0.95 (SD = 1.77). In a previous study involving 15-year-old adolescents, the prevalence of caries experience on anterior teeth was 17.6%^19^. A quantitative measure was used in the present study, which is more sensitive to the severity of dental caries since such a measure provides data on the number of teeth affected by caries and not simply the absence or presence of the condition, making the data more robust. A study conducted with 12-year-old adolescents found an average of 1.31 (SD = 2.71) teeth with untreated caries, differing from the present investigation by considering both the anterior and posterior regions^7^. Differences in the prevalence of dental caries between the anterior and posterior regions may be explained by the greater ease of cleaning the anterior teeth. Untreated caries on anterior teeth is an indication of high severity of this oral disease and is a more sensitive indicator of limited access to dental services and hygiene practices^6,19^.

The number of anterior teeth with untreated caries was greater in male adolescents than females. This finding may be explained by the fact that female adolescents generally have healthier oral hygiene practices, such as a greater frequency of brushing^20^ and more visits to the dentist^21^, in comparison to male adolescents. Poorer oral health behaviors in males may reflect cultural stereotypes that associate the search for health care with the female sex, attributing greater resistance to health-related events to the male sex^22^. It is also possible that oral health strategies are not directed specifically to male adolescents, whose health needs are not yet seen as a priority^23^. Based on these findings, oral health education actions should consider these individuals to reverse this behavior and strengthen preventive habits.

Adolescents with inadequate and marginal OHL had more caries than those with adequate OHL. Previous studies with this population demonstrated similar results considering untreated caries in anterior and posterior regions^10,11^, suggesting that low OHL in adolescents is an important issue to address since this determinant affects oral health outcomes in this population^7^. Therefore, appropriate health strategies should be formulated to improve communication between dentists and these individuals^15^. A population-based study conducted in Brazil with children reported similar findings^24^, underscoring the importance of drafting a national plan to strengthen literacy as a social determinant of health^25^

Another factor associated with the number of anterior teeth with untreated caries in adolescents was the absence of a private health insurance plan. Previous studies confirm these findings, reporting a greater frequency of dental caries in adolescents who use public services compared to those who use private services^11,26^. These findings may be related to the greater access to information regarding the prevention of oral problems at private services^27^, which may be related to the greater availability of time on the part of the dentist due to the lower demand for care in comparison to public dental services^11^.

The school context exerted an influence on the occurrence of untreated caries in the adolescents aging 15 to 19. Few studies have investigated the influence of the school setting on oral health in this age group^28,29^ and, to the best of our knowledge, no previous studies have evaluated the contextual factors of the school analyzed in the present investigation. Adolescents enrolled at schools with a larger average number of students per class had more than twice as many anterior teeth with untreated caries compared to those enrolled at schools with a smaller average number of students per class. The school setting is important to the learning process of adolescents as well as oral health practices^27^. A previous study involving 12-year-old adolescents found that individuals enrolled at public schools and with a higher failure rate (students that held back two or more consecutive times) had a greater number of dental caries in general^7^. Thus, a school environment that does not contribute to learning seems to favor the occurrence of dental caries^30^, likely due to the difficulty in implementing and perpetuating oral health prevention in this environment. Large schools (measured by the number of students) hinder a personalized teacher-student relationship by placing greater demands on teachers, with a negative impact on the educational process^31^. These findings underscore the importance of reorganizing the teaching environment so that it is more productive, which may influence the prevention of dental caries on anterior teeth in adolescents.

Another school contextual factor associated with the number of anterior teeth with untreated caries was the number of public dental services available in the district where the school was located. The greater availability of services may have contributed to a lower number of teeth with untreated caries in the present sample. In a study conducted in Brazil with five-year-old children, the number of oral health teams in the administrative district of the school was not associated with cavitated carious lesions^32^. This difference may be explained by the low frequency of seeking dental services in early childhood when children are more dependent on their parents/guardians^33^. In contrast, adolescents have more autonomy regarding their health, and are more likely to perceive a treatment need when it affects dental esthetics^5^. Therefore, public policies should be implemented to expand the number of dental services, especially around schools in areas with social deprivation, where dental caries have a greater social impact.

The average income of the district in which the school was located was associated with the number of anterior teeth with untreated caries. Enrollment at schools located in districts with a higher average income was a protection factor against the occurrence of dental caries on anterior teeth. Previous findings have demonstrated the effect of a low socioeconomic status of the school context on dental caries^30^. The protective effect of the average income of the school district is likely explained by the limited access to oral hygiene materials and difficult access to education in areas of social deprivation. Moreover, since the adolescents share a similar socioeconomic situation, the school environment strongly influence health practices^13^.

The cross-sectional design constitutes a limitation of the present study due to the impossibility of establishing causality between variables. However, the study involved a representative sample, which strengthens its external validity, and multilevel analysis was performed, which enables the evaluation of contextual factors that exert an influence on untreated caries on anterior teeth in adolescents. Moreover, the training and calibration of the examiners, the conduction of a pilot study to test the methods and the use of validated instruments strengthen the internal validity of this study.

Knowledge regarding individual and contextual factors associated with dental caries on anterior teeth is important and can contribute to more effective health policies directed to the prevention of oral health inequalities in adolescents, especially in areas of social deprivation. Thus, the present findings can assist in the creation of healthier school environments that prioritize oral health education measures.

In conclusion, sociodemographic factors, having private health insurance, oral health literacy, and school context exerted an influence on the occurrence of untreated dental caries in adolescents aging 15 to 19 in the present study.
